# Brain tuberculoma: a 52-year-old woman case report

**DOI:** 10.1099/acmi.0.000634.v4

**Published:** 2023-10-16

**Authors:** Gokul S. Shankar, Sreeja Nair, Teena Jacob, Mercy John Idikula

**Affiliations:** ^1^​ Department of Microbiology, Pushpagiri Institute of Medical Sciences & Research Centre, Thiruvalla, Kerala, India

**Keywords:** brain biopsy, CSF, liquid Culture, *Mycobacterium tuberculosis*, tuberculoma

## Abstract

**Introduction.:**

One of the most serious extrapulmonary type of tuberculosis that affects people under the age of 40 is brain tuberculoma. They are space-occupying masses of granulomatous tissue that result from hematogenous spread from a distant focus of tuberculous infection by *Mycobacterium tuberculosis *. Symptoms and radiologic features being nonspecific usually leads to misdiagnosis and mimics a variety of other infectious diseases. Anti-tubercular drugs are essential for the successful treatment of cerebral tuberculomas.

**Case Report.:**

The authors present a case report of a 52-year-old diabetic woman, who presented to the Emergency Department of a tertiary care hospital and was diagnosed with brain tuberculomas with a brain biopsy. Brain tuberculomas are rare and could be overlooked. Therefore, this is an important consideration in cases with higher suspicions, given the rapid decline in patient condition.

**Conclusion.:**

Due to their rarity, ambiguous symptoms, and radiographic characteristics, intracranial tuberculomas continue to provide a clinical challenge and must always be considered in the differential diagnosis of cerebral space occupying lesions. As CSF may not yield positivity for both CBNAAT and smear examination, a brain biopsy specimen for culture should always be kept in mind for detecting tuberculoma and initiating anti-tubercular treatment at the earliest.

## Data Summary

No data was generated during this research or is required for the work to be reproduced.

## Introduction

TB is one of the top causes of death due to a single infectious pathogen [1]. In 2021, 1.6 million people died from tuberculosis (including 187 000 HIV patients). TB is the 13th largest cause of mortality worldwide and the second leading infectious killer after COVID-19 (behind HIV and AIDS) [1].

According to the most recent WHO study from 2022, *M. tb* infection affects approximately one-fourth of the global population [1]. The 30 countries with a high TB burden, accounted for 87 % of new TB infections in 2020. Eight nations, which include India, Indonesia, China, Philippines, Pakistan, Nigeria, Bangladesh and the Democratic Republic of the Congo, accounted for more than two-thirds of the global total [1]. Tuberculoma of the brain is one of the most serious extrapulmonary types that afflict people under the age of 40 [2]. Tuberculomas account for approximately 33 % of cerebral space-occupying lesions in developing-country individuals [3]. Nonspecific symptoms and radiological characteristics mirror a range of different infectious, leading to misdiagnosis [2].

The involvement of the CNS is one of the most serious signs of EPTB. The CNS is the sixth to seventh most commonly infected tissue by EPTB (2 –15 %), with a higher frequency in AIDS patients [4]. CNS tuberculosis accounts for about 1 % of all tuberculosis cases and is associated with a significant mortality and morbidity rate [5].

Patients with HIV infection are five times more likely to develop CNS tuberculosis [6]. The most typical symptom of CNS tuberculosis is tuberculous meningitis, which is followed by tuberculous abscess and cerebral tuberculoma. According to Salaskar *et al.,* TB causes about 4 % of central nervous system lesions in industrialized nations and 15–30 % in impoverished countries [7]. Immune suppression is seen commonly in CNS TB patients, which includes cancer, chemotherapy, HIV, diabetes mellitus, malnutrition, chronic renal failure, etc [8].

According to estimates, 50 % of cases of disseminated TB and one out of every 300 untreated cases of pulmonary TB acquire TB in the brain parenchyma [9]. Studies have shown that 6 to 12 months prior to receiving a CNS tuberculosis diagnosis, over 75 % of patients experienced pulmonary tuberculosis. However, between 25 and 30% of patients with brain TB do not have pulmonary TB [10].

The delay of an early diagnosis and treatment can be explained by the absence of a previous history of tuberculosis in more than half of the patients, the indistinct initial clinical presentation, and the prevalent radiological characteristics [11]. Tuberculomas can also mimic other entities, including Glioblastoma, brain metastasis, intracranial haemorrhage, and abscesses, which can be associated with calcifications that produce the ‘target sign’ that suggests reactivation or dormant tuberculosis [9]. The most common image of tuberculoma is a ring-enhancing lesion due to the absence of blood supply in the caseous necrosis centre within the tuberculoma [9].

However, a number of other illnesses might potentially result in the same imaging findings. Some of them include cerebral venous thrombosis, toxoplasmosis, bacterial abscesses, cryptococcosis, syphilis, sarcoidosis, inflammatory or vascular abnormalities, and various brain tumours (including glioblastomas, low-grade gliomas, lymphomas and brain metastases) [9].

Brain tuberculomas can present as a subacute or chronic condition that lasts weeks to months and is more common in immunocompromised patients. The clinical course may be asymptomatic in patients with isolated or sparse parenchymal lesions, but if these lesions are numerous or large, symptoms like fever, vomiting, headaches, focal neurological deficits, seizures, hydrocephalus, meningeal irritation signs and intracranial hypertension with papilledema are more likely [9]. In 1.8 % of TB patients, the CNS is impacted, and in 24 % of cases, brain tuberculoma is the only symptom seen [12].

Smear microscopy, CBNAAT, and culture are the most regularly used microbiological diagnostic procedures, with sensitivities ranging from 20–55 % for smear, 40 % for CBNAAT, and 60 % for culture, respectively [13].

When brain tuberculoma is the only lesion, CSF investigations are often normal; however, when intracranial pressure is elevated, the white cell count may be elevated, favouring the secondary ischaemic brain parenchyma [9].

Although brain tuberculoma can be identified by PCR for CSF, it might not be helpful for an immediate diagnosis and course of treatment [14].

More than 85 % of cases of tuberculomas can be cured if therapy is started early. Isoniazid, Rifampicin, Ethambutol and Pyrazinamide are used as the first line of anti-tubercular therapy for 2 months, followed by dual therapy (Isoniazid and Rifampicin). When there is significant cerebral oedema or concomitant meningeal involvement, corticosteroid treatment is typically used [15]. CNS TB has a worse prognosis than pulmonary TB, if treatment is started late [16].

## Case report

A 52-year-old woman with, a known case of type 2 diabetes mellitus, systemic hypertension, dyslipidaemia, hypothyroidism and chronic kidney disease presented to the Emergency Department of a tertiary care hospital with complaints of disoriented talk.

Following disoriented talk in the Emergency Department, she was subjected to CT brain, which showed ICH and later shifted to MICU.

### General examination

At the time of admission to the medical ICU, she was drowsy with a GCS (E2 V1 M4) without any focal neurological deficits, temperature of 104 °C, tachycardia, and hypotension requiring minimum noradrenaline support.

She was intubated because of low GCS and antibiotic Inj. Piperacillin Tazobactam 2.25 mg BD IV was started empirically after obtaining a blood sample for blood culture.

### Initial radiological findings

MRI of the brain to reassess the ICH bleeding in CT brain showed multiple ring-enhancing lesions.

### Lab investigations

Complete blood count showed WBC-10,800/mm^3^, Procalcitonin- 1.9. Urine routine showed numerous pus cells.

Urine culture showed the presence of *Klebsiella pneumoniae* (10^6^ c.f.u. ml^−1^) and was sensitive to Inj. Meropenem. Antibiotics were escalated to Inj. Meropenem 1 gm IV TID × 7 days and Inj. Vancomycin 1 gm IV BD × 5 days.

Her GCS improved (E4VTM5), hemodynamics stabilized and the patient was extubated within the next 3 days.

A differential diagnosis of septic emboli/cerebral vessel vasculitis/tuberculous encephalitis was considered and planned for trans oesophageal echocardiography (TEE), peripheral blood smear, autoantibody screen, CSF study, India ink staining, Gram staining and HIV screening. Due to persistent fever, a repeat blood culture was sent. All the aforesaid investigations were insignificant except for CSF study, which showed a total count of 128 cells, polymorphs – 12, lymphocytes 88 and protein 119 g dl^−1^, glucose 90 mg dl^−1^. GeneXpert was done on the CSF sample and was found negative.

The patient continued to have fever spikes and was started in NIV support. In view of hypovolemia, desaturation, hypotension, patient was re-intubated. Tab Fluconazole 400 mg was added due to elevated counts (12000 /mm^3^). CT chest was done, which showed mediastinal lymph nodes. Endotracheal secretions for ZN staining and CBNAAT were negative.

The patient showed a further drop in GCS and started on anti-tubercular along with steroids, since repeat MRI brain and spine showed multiple tuberculoma with no resolution and, mild obstructive hydrocephalus of the third ventricle.

After considering the radiological imaging, deteriorating condition, and no confirmatory diagnosis being made, the patient underwent a navigation brain biopsy and samples were sent for bacterial, fungal and mycobacterial culture by MICRO MGIT. Blood C/S grew *Sternotrophomonas maltophila* and was escalated to Inj. Colistin 1 million IU BD and T. Cotrimoxazole 480 mg BD × 5 days.

**Fig. 1.**
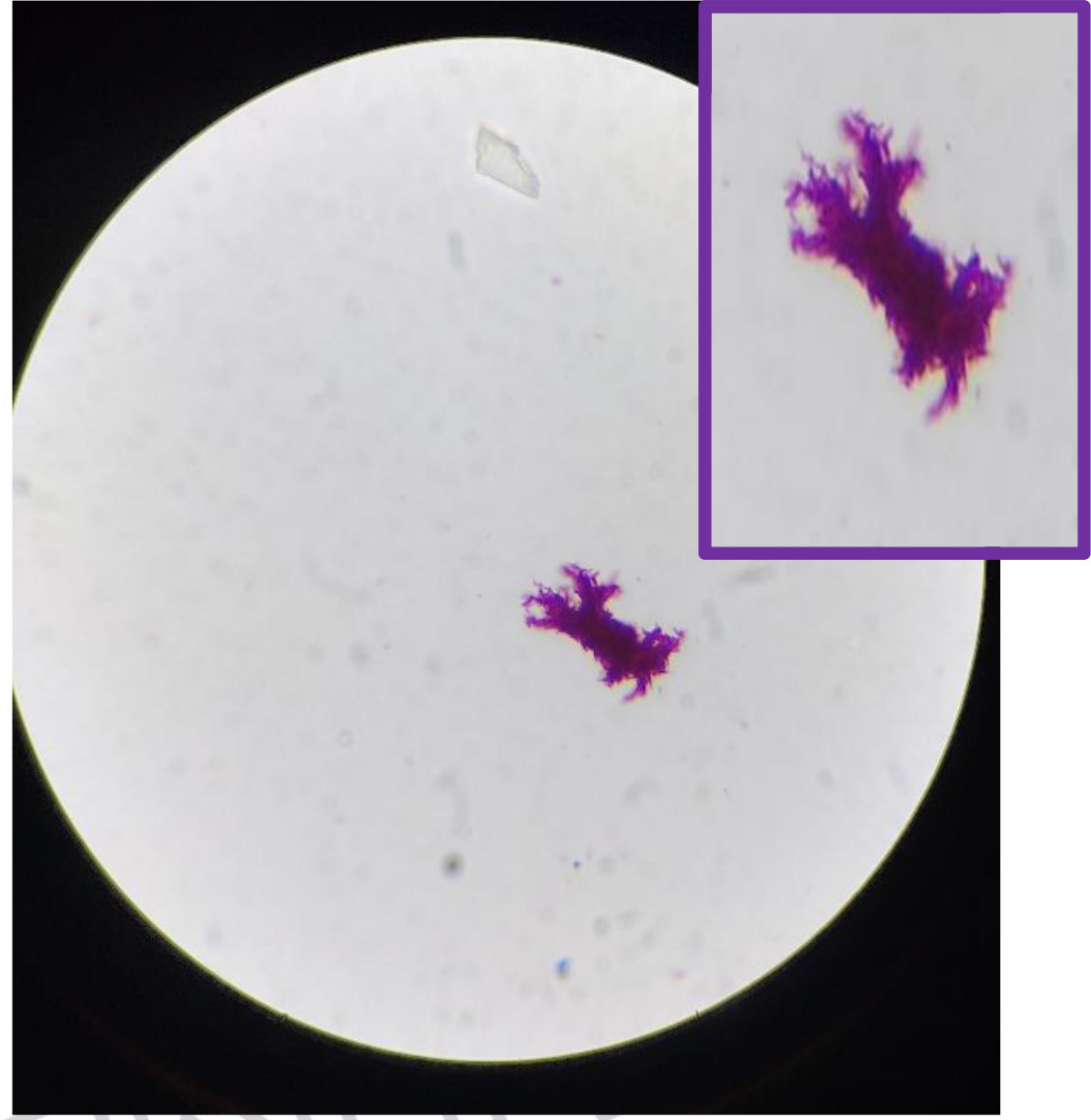
*M. tb* showing cording in ZN smear of brain biopsy culture.

**Fig. 2.**
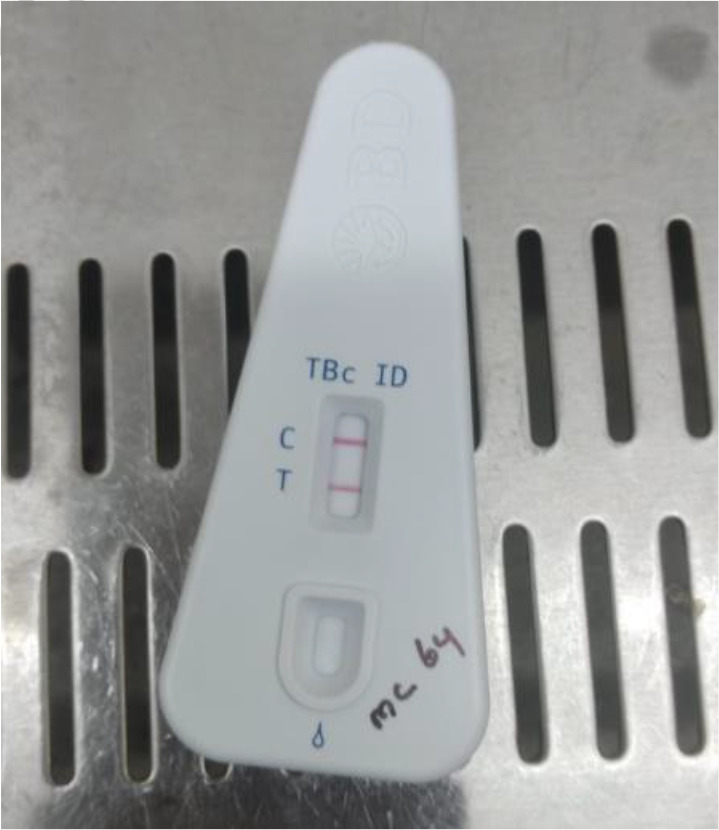
MPT 64 Antigen Kit (BD Diagnostics) showing positive for *M. tb*

The patient's condition continued to deteriorate and chances of septic encephalopathy, intracranial haemorrhage and cerebral oedema due to inflammation from TB encephalitis were considered. A repeat MRI brain showed dilated third and fourth ventricles for which the patient was shifted to the neurosurgery ICU and an extra ventricular drain was placed. The patient with no signs of clinical improvement finally succumbed to death (Table 1). Brain biopsy was negative for fungal and bacterial culture but 6 days after her demise, the mycobacterial culture of the brain biopsy grew *Mycobacterium tuberculosis* complex. (Time of positivity, 12 days.) This was confirmed by ZN smear (Fig. 1) and MPT 64 Antigen Kit (BD Diagnostics) (Fig. 2).

**Table 1.** Sequence of investigations and findings

DATE	Procedures / Findings	Investigations
HD^*^ 1	CT brain→ subarachnoid haemorrhage	
**HD 3**	MRI brain→ multiple brain enhancing lesions (Fig. 3)	
**HD 6**	Trans oesophageal echocardiography→no vegetation	Lumbar puncture: CSF study→almost normal ZN smear→ AFB not seen ANA screen→ negative HIV→ Nonreactive Peripheral blood smear→normal study
**HD 7**	CT chest→mediastinal lymph nodes	
**HD 9**		ZN smear(endotracheal secretion)→No AFB seen CBNAAT→ negative
**HD 12**	Repeat MRI→multiple tuberculoma and mild obstructive hydrocephalus of the third ventricle	ATT started
**HD 14**	Repeat MRI→ dilated third and fourth ventricle with tuberculoma	
**HD 15**	Navigation brain biopsy	Fungal culture→ no growth Bacterial culture→ no growth Mycobacterial culture→*M. tb* grown in MGIT (12 days to positivity) ZN smear→ AFB cording (Fig. 1) LJ culture→ negative after 42 days of incubation
**HD 21**	Succumbed to death	

*HD, hospital day.

**Fig. 3.**
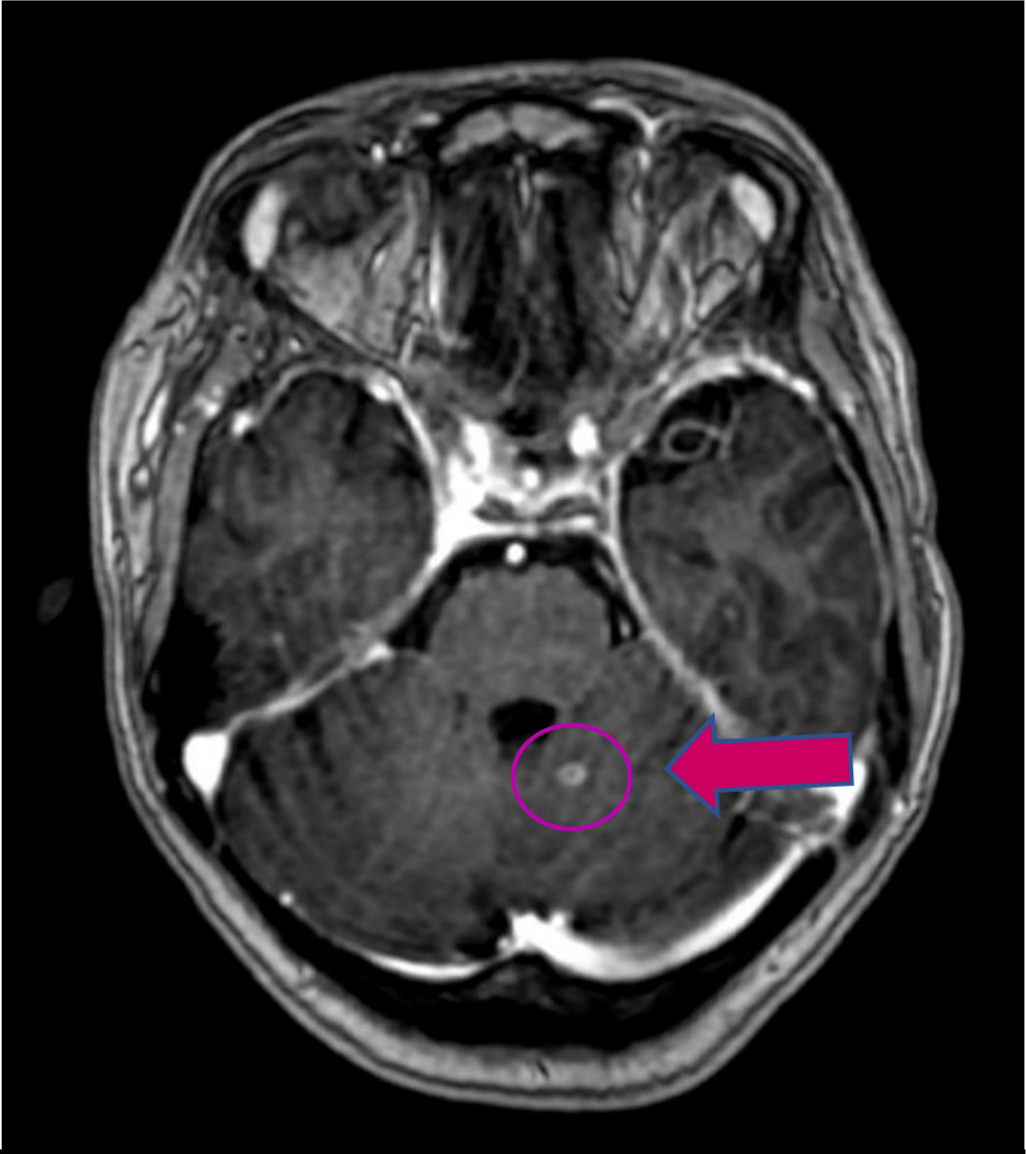
Red arrow showing brain enhancing lesion in MRI.

## Discussion

Tuberculomas are clumps of caseous tissue in the brain that can occur with or without meningitis [17]. It accounts for 20–30 % of all intracranial tumours and can also result in brain abscesses in poor host defence mechanisms [2]. The presentations are often clinically silent and results in calcifications that are single/multiple therefore requiring multiple investigations and special tests.

The initial clinical presentations and radiological investigations were not in favour of tuberculosis. Considering India, an endemic country for tuberculosis, the poor prognosis of patient and multiple ring enhancing lesions in MRI, the differential diagnosis was narrowed down more towards tuberculosis. Further, most of the other conventional microbiological investigations like ZN staining, India ink, Gram-staining were all negative thus ruling out most of the other infectious causes. This could be due to the paucibacillary nature of the disease.

Tuberculomas and neurocysticercosis lesions resemble each other in many aspects on contrast enhanced CT and contrast MRI. Tuberculomas are generally solitary, but multiple nodular ring-like enhancing lesions similar to NCC are also found in 15–34 % of CNS TB, which often confuse the diagnosis [18]. In a study by Sonia Ahlawat *et al.,* I-PCR based on ESAT-6 detection in CSF was more sensitive in comparison to conventional microbiological (smear/culture) and molecular-PCR tests for the diagnosis of cerebral tuberculomas [19]. I-PCR has been documented to be a rapid, robust and highly sensitive method for the detection of mycobacterial antigens up to picogram levels from sputum and pleural fluids of Tb patients [19].

The CSF sample was negative for ZN staining in the present case. This is similar to the study done by Talamás *et al.* on 11 patient samples in cases of brain stem tuberculoma [20]. The study also showed that when brain parenchyma was involved, ZN staining of CSF for AFB (sensitivity 70 %, specificity 97.1 %) and CSF culture for *M. tb* were negative [20]. The limited sensitivity of CSF smear examination is due to the small number of bacilli present in the CSF, the small volume of CSF taken at the tap for evaluation most of the time, and the fraction examined using an oil immersion lens. This is similar to the study done for the detection of tubercular meningitis in GMC, Srinagar by Iqbal et al., where the sensitivity of CSF was only 2.6 % [21], and studies by Marais S *et al.,* reported the sensitivity ranging from 2–30 % in CSF by smear microscopy [22].

Smear microscopy always results in low sensitivity and requires the presence of 10^3^ bacilli/ml in the sample to allow detection and its sensitivity is also often low in EPTB cases due to the paucibacillary nature of specimens [23]. In a study by Khan *et al.,* they reported sensitivities of 37.5% and 0 % in confirmed and suspected OATB cases respectively, for detecting AFB by smear microscopy [23].

The CSF showed predominant polymorphs, which otherwise showed a normal study in this case. Therefore, it is important to analyse the clinical course and evaluate predisposing factors capable of suppressing the immune system, which favours acquired opportunistic infections. When brain tuberculoma is the only lesion, CSF analysis might be normal; however, due to the increased intracranial pressure, the white cell count might be elevated favoured by the secondary ischaemic brain parenchyma [9].

Due to the lack of blood supply in the caseous necrosis centre within the tuberculoma, the most common visual representation of the disease is a ring-enhancing lesion [21]. According to a case report of a brain tuberculoma case in a 15-year-old girl by Saleh *et al.* the CT findings in that case, showed multiple ring-enhancing lesions but the finding based on CT alone was presumptive [2]. In cases of multiple brain tuberculomas, brain biopsy is the most reliable method for diagnosis, and the case’s biopsy results strongly confirmed this as the correct diagnosis [2]. This was similar to the clinical manifestations in the present case where the CT findings showed similar multiple ring-enhancing lesions and required a brain biopsy culture for the final confirmation.

Liquid culture by Micro MGIT grew *M. tb* even though solid LJ culture did not grow *M. tb* after 42 days of incubation. Time to positivity for *M. tb* culture was shorter with MGIT for smear-negative specimen (12 days). This was comparable to research done by Chihota *et al*. in 2010 who demonstrated that MGIT provides higher yield and faster outcomes at a relatively high cost compared to solid culture [24].

In India, Selvapandian *et al.* demonstrated that CT scans have 100 % sensitivity and 86 % specificity in detecting tuberculoma. However, even in a high-incidence group, the positive predictive value can be as low as 33 % [25]. Biopsy is also useful when imaging investigations demonstrate the progression of brain lesions, such as a paradoxical reaction to anti-tuberculosis drug therapy, or when the patient is afflicted with drug-resistant TB or is noncompliant [25]. The brain biopsy was considered in the current case after considering all the infectious causes, poor response to medical therapy, and radiological imaging to get a confirmatory diagnosis.

The GeneXpert MTB/RIF assay (Xpert), a WHO-approved semi-automated technology, has transformed TBM diagnosis by detecting *M. tb* and rifampicin resistance directly from CSF samples within 2 h [26]. Xpert Ultra exhibited a sensitivity of 72.05 % and a specificity of 100 % in the overall diagnosis of TBM in the study by Sharma *et al.,* of Xpert MTB/RIF ultra for the diagnosis of tuberculous meningitis [26].CBNAAT for the CSF sample was also negative in this case indicating the need for a holistic approach, which sometimes requires a brain biopsy culture when a differential diagnosis is made.

According to a study by Sehgal *et al.,* on comparison of protein B PCR with IS6110 PCR for diagnosis of tuberculous meningitis patients, showed that molecular tools such as PCR have the potential to increase the clinician’s ability to diagnose tuberculous meningitis. Pab(protein b antigen) PCR is a rapid and reliable method for diagnosing tubercular meningitis in routine microbiology laboratories. It had the best sensitivity (82 %) when compared to other tests, including PCR IS6110, and found four cases that IS6110 missed. As a result, Pab PCR can be employed as a simple, inexpensive, quick and dependable diagnostic to increase the clinician’s capacity to detect TBM [27].

PCR for CSF can be useful to diagnose brain tuberculoma; however, it might not be useful for a rapid diagnosis and treatment, which amounts to not sending the sample for PCR in the current case considering the low sensitivity and specificity of CSF sample for PCR and lack of available facility in our hospital [14].

The treatment of brain tuberculomas is primarily pharmacological, using several first-line anti-tubercular medicines. Some authors advocate for empirical medical therapy without histological confirmation, whereas others believe that such medication should be administered until a confirmatory diagnosis is made [28]. The anti-tubercular medicines and duration of treatment for brain tuberculoma are not well understood [29]. In this case, the patient was started on anti-tubercular drugs but they were withdrawn due to the patient’s noncompliance.

## Conclusion

Intracranial tuberculomas are extremely rare and dangerous forms of extrapulmonary tuberculosis. It is caused by *M. tb* hematogenous spread in immunocompromised young people, resulting in severe mortality and morbidity. Intracranial tuberculomas remain a clinical challenge due to their rarity, cryptic symptoms and radiological abnormalities. It should be included in the differential diagnosis of lesions occupying cerebral space. A brain biopsy specimen for culture and/or CBNAAT should be considered for diagnosing tuberculomas and starting ATT if CSF is negative on AFB smear and CBNAAT.

We believe this report can support healthcare providers to consider tuberculoma in the differential diagnosis of intracranial lesions in patients with such clinical-radiological characteristics living in areas of high tuberculosis incidence.

The limitation of this case report is that PCR was not utilized for detection due to the lack of availability of TB-PCR in our hospital and also accounting for the low sensitivity and specificity of CSF sample for molecular diagnosis. In a study by Khan *et al.,* the sensitivities attained by multi-targeted LAMP in total OATB cases were significantly higher [the sensitivities of 100 and 82.4 % were obtained in confirmed (*n*=10) and suspected (*n*=57) OATB cases, respectively, by multi-targeted LAMP with a specificity of 96.9 % (*n*=33)] than multiplex PCR (mpt64 +pstS1) and GeneXpert assay for early detection of OATB cases, which further supports the limitation of PCR in the detection of EPTB [30].
